# Large-Scale Brain Simulation and Disorders of Consciousness. Mapping Technical and Conceptual Issues

**DOI:** 10.3389/fpsyg.2018.00585

**Published:** 2018-04-24

**Authors:** Michele Farisco, Jeanette H. Kotaleski, Kathinka Evers

**Affiliations:** ^1^Centre for Research Ethics and Bioethics, Uppsala University, Uppsala, Sweden; ^2^Science and Society Unit, Biogem Genetic Research Centre, Ariano Irpino (AV), Italy; ^3^Science for Life Laboratory, School of Computer Science and Communication, KTH Royal Institute of Technology, Stockholm, Sweden; ^4^Department of Neuroscience, Karolinska Institute, Solna, Sweden

**Keywords:** consciousness, consciousness disorders, brain modeling, neuroethics, brain simulation

## Abstract

Modeling and simulations have gained a leading position in contemporary attempts to describe, explain, and quantitatively predict the human brain’s operations. Computer models are highly sophisticated tools developed to achieve an integrated knowledge of the brain with the aim of overcoming the actual fragmentation resulting from different neuroscientific approaches. In this paper we investigate the plausibility of simulation technologies for emulation of consciousness and the potential clinical impact of large-scale brain simulation on the assessment and care of disorders of consciousness (DOCs), e.g., Coma, Vegetative State/Unresponsive Wakefulness Syndrome, Minimally Conscious State. Notwithstanding their technical limitations, we suggest that simulation technologies may offer new solutions to old practical problems, particularly in clinical contexts. We take DOCs as an illustrative case, arguing that the simulation of neural correlates of consciousness is potentially useful for improving treatments of patients with DOCs.

## Neuronal Underpinnings of Consciousness

Even if “consciousness” as such and the brain-mind relation in general are highly debated concepts within both neuroscientific and philosophical communities (Metzinger, 1995; Chalmers, 1996; Block et al., 1997; Revonsuo, 2006; Dehaene, 2014; Facco et al., 2015; Laureys, 2015; Tononi et al., 2016), most recognize the relevance of the empirical findings identifying so called “neural correlates of consciousness” (NCC), i.e., a set of neuronal structures and functions correlating with conscious phenomena. Since their formal introduction in the scientific debate at the beginning of 90s (Crick and Koch, 1990), NCC have been widely scrutinized from both conceptual and empirical points of view (Metzinger, 2000b; Koch et al., 2016). Conceptually, NCC are defined by Chalmers as minimal neuronal activations necessary for consciousness (Chalmers, 2000). Such a general definition has been widely accepted in both philosophical and empirical contexts, even though the need for a more stringent conceptual definition of NCC has recently been argued for (Fink, 2016).

More specifically NCC can be depicted in two basic ways: either as referring to a general, global state of consciousness, i.e., as neural correlates that mark the difference between being and not being conscious; or as referring to particular contents of consciousness, i.e., as neural correlates that are sufficient for a specific object to enter consciousness (Chalmers, 2000; Overgaard, 2017). Actually Chalmers proposes an even more detailed differentiation: he distinguishes between NCC of creature consciousness (“the property a creature has when it is conscious, and lacks when it is not conscious”), NCC of background consciousness (“an overall state of consciousness such as being awake, being asleep, dreaming, being under hypnosis, and so on”), which is finer-grained than creature consciousness but still not defined in terms of specific contents, and NCC of consciousness as fine-grained specific states often individuated by their contents (Chalmers, 2000). For the sake of our argument, the first two types of NCC can be conflated into one (NCC of state-consciousness), as distinguished from NCC of specific conscious contents.

Chalmers provides an overall definition of both the NCC above. NCC of state-consciousness: “An NCC is a minimal neural system N such that there is a mapping from states of N to states of consciousness, where a given state of N is sufficient, under conditions C, for the corresponding state of consciousness” (Chalmers, 2000) (p. 31). NCC of content-consciousness: “An NCC (for content) is a minimal neural representational system N such that representation of a content in N is sufficient, under conditions C, for representation of that content in consciousness” (Chalmers, 2000) (p. 31). It is worth noting that NCC are qualified as sufficient but not necessary because otherwise the definition would be too strong (in fact there might be more than one NCC of a given conscious state). Moreover NCC are qualified as “minimally sufficient” rather than simply “sufficient” in order to avoid any irrelevant part of the brain not directly involved in consciousness (otherwise all the brain would count as NCC). In fact Chalmers distinguishes between total NCC (comprising the totality of physical processes absolutely required for a conscious state) and core NCC (comprising only the core processes correlated with the target conscious state).

Importantly, Chalmers specifies that being a *correlate* of consciousness, it does not follow that NCC is only dedicated to consciousness, or that NCC is the most responsible for the generation of consciousness, or that NCC is an explanation of consciousness. This would go too far, too fast. Science explores correlation, which is not necessarily the same as explanation. This point is stressed also by Metzinger, who outlines that even if fine-grained correlations between brain states and consciousness states are established, several theoretical options remain open (Metzinger, 2000a). In fact correlation as such is compatible with dualism (e.g., causal interaction between two ontologically distinct domains), with epiphenomenalism (i.e., one-way causation from neuronal to phenomenal), and with the absence of any causal relationship between the neuronal and phenomenal levels because both are dependent on events re-establishing the observed correlation or because both are different aspects of the same underlying reality. Thus Metzinger rightly concludes that correlation alone is not sufficient for a theory of consciousness (Metzinger, 2000a).

Notwithstanding this legitimate caution, the above-summarized description of NCC has been widely used in scientific research. Regarding content-specific NCC, there has been a debate among neuroscientists whether to identify them with systems in the prefrontal cortex (late activations) or with systems in occipital/parietal cortices (early activations) (Overgaard, 2017). The increasingly accepted view is that the latter hypothesis is the more likely, while late activation in prefrontal cortex would be correlate of metacognition, attention, task execution, monitoring, and reporting rather than of consciousness (Aru et al., 2012; Koch et al., 2016).

According to the most recent research in the field, the same is true for NCC of state-consciousness. Even in this case the best current anatomical candidates are localized in a temporo-parietal-occipital zone of the posterior cerebral cortex (Koch et al., 2016).

In addition to NCC, background conditions for consciousness are recognized as important. Particularly, neuronal populations within subcortical regions, like the brainstem, hypothalamus and basal forebrain, provide an important background condition for consciousness facilitating effective interactions among cortical areas (Parvizi and Damasio, 2001). Yet these background conditions might be not necessary for consciousness if an appropriate subset of cortical regions has sufficient intrinsic activation (Nir et al., 2011; Koch et al., 2016). Accordingly, the role of basal ganglia, claustrum and thalamus in enabling consciousness is still debated.

Besides dualistic positions, which state that consciousness and brain are different in nature (Robinson, 2017), both philosophical and neuroscientific communities recognize NCC as important or even critical for consciousness. As already said, correlation is not self-explanatory or sufficient to explain why there is consciousness rather than no-consciousness correlated to the target neuronal activity. This is one of many hot issues in conceptual analysis of NCC (Revonsuo, 2006). Problems like this are far from solved and promise to engage massive speculative work in the years to come. While we acknowledge the conceptual importance of these issues, in this paper we do not tackle them specifically. Rather it is worth focusing a bit on the clinical sides of these problems.

In clinical practice, even if the abovementioned conceptual issues are relevant, priorities are practical: clinicians need to make decisions on how to treat patients. In borderline cases, such as DOCs, measuring residual consciousness is of central importance to make the most appropriate decisions. For doing so clinicians can focus only on residual brain activity, particularly in areas recognized as correlating with specific conscious activities (i.e., state- or content-specific consciousness).

For illustrative purposes, we can consider the identified neuronal underpinnings of two components of consciousness that are considered very important in clinical contexts, with particular regard to DOCs: wakefulness (or level of consciousness) and awareness (or consciousness’ content) (Laureys, 2005; Laureys and Schiff, 2012).

Neuroimaging experiments have tried to define accurate biomarkers of wakefulness and awareness in unresponsive wakefulness syndrome/vegetative states (UWS/VS) and in minimally conscious states (MCS), two disorders often very difficult to disentangle (Schnakers et al., 2009).

To illustrate, the functional and structural integrity of ascending ponto-mesodiencephalic reticular pathways and widespread thalamocortical projections has been shown as essential for igniting and maintaining the level of consciousness (i.e., wakefulness) (Steriade, 1996; Laureys et al., 2004).

Regarding awareness, it seems that the relationship between global levels of brain function (e.g., global metabolic activity) and the presence or absence of awareness is not absolute. In other words, metabolic activity in some specific areas is likely correlated with awareness (Laureys, 2005). In fact, it has emerged that, besides the activation of low-level specialized cortices (Boly et al., 2012), awareness requires the activation of a wide frontoparietal network, including lateral and medial frontal regions bilaterally, parieto-temporal and posterior parietal areas bilaterally, posterior cingulate and precuneal cortices (Laureys et al., 1999). Equally correlated with awareness are the connections within the frontoparietal network and between the frontoparietal network and the thalamus (cortico-cortical and cortico-thalamo-cortical connectivity) (Laureys et al., 2000), and the general level of functional integrity within the nested hierarchy of neuronal assemblies and ever increasing complex spatial-temporal structures of synchronized neuronal assemblies (Fingelkurts et al., 2012b). Significantly, different networks for internal or self awareness (i.e., relative to the self) and for external or sensory awareness (i.e., relative to the external world) have been identified (midline fronto-parietal and lateral fronto-parietal networks, respectively) (Vanhaudenhuyse et al., 2011; Fingelkurts et al., 2012a).

Recent empirical findings show that also the intrinsic activity of the brain (i.e., the brain activity independent from external stimulation) and the resting state brain activity (i.e., the brain activity that increases in absence of stimuli) are correlated with consciousness (Boly et al., 2008; Northoff, 2013b). Scientific evidence suggests that the connectivity within and between the midline default mode network (DMN) and the lateral frontoparietal cortices is correlated to conscious perception (Noirhomme et al., 2010): the conscious perception of a stimulus is associated with whole-brain dynamic alterations in functional connectivity (i.e., in the connectivity between brain regions sharing functional properties).

Furthermore, it has recently been shown that the default mode activity of the brain expresses a correlation between subjective ‘internal’ self-related thoughts with activity in midline cortical structures and ‘external’ sensory perceptions with lateral frontoparietal activity (Vanhaudenhuyse et al., 2011). Moreover, the DMN connectivity differentiates between different DOCs (Boly et al., 2009; Vanhaudenhuyse et al., 2010; Demertzi et al., 2015). Furthermore, it has been shown that the integrity of the frontal subnet of the DMN is capable to predict future recovery of consciousness in UWS/VS (Fingelkurts et al., 2016).

Accordingly, there is broad agreement that self-consciousness is correlated with the functional connectivity within the DMN and between the DMN and the thalamus (Vanhaudenhuyse et al., 2010; Huang et al., 2014). DOCs show an impairment of both functional and effective (i.e., causal) connectivity (Rosanova et al., 2012), with a resulting neural activity that seems to be more local, simple and short-lived than healthy conditions (Boly et al., 2007; Northoff, 2013a, 2014). Yet other findings also show a pathological hyperconnectivity between the DMN and external areas, such as the subcortical limbic system, including the orbitofrontal cortex and the insula (Di Perri et al., 2013).

A characteristic of the resting state, relevant for modulating conscious processing, is a switch between the dominance of DMN – linked to internal or self-awareness – and the bilateral frontoparietal network, which is linked to external or environmental awareness (Cavaliere et al., 2016). These anticorrelated patterns have been shown to be correlated to mentation (Vanhaudenhuyse et al., 2011) and, since their anticorrelation is reduced in DOCs (Demertzi et al., 2015), it is likely that they are relevant for the phenomenological complexity of consciousness as well (Demertzi et al., 2013).

## Large-Scale Brain Simulation

Two kinds of simulation are possible: a global or large-scale simulation and a discrete or subsystem simulation. More specifically, a large-scale simulation of the brain can in principle be implemented in two ways: as a simulation of the whole brain at different scales simultaneously in runtime or as a simulation of the whole brain at specific scales and levels. The discrete simulation is already abundantly used as a research tool in neuroscience, also with the specific aim to validate theories of conscious access (Dehaene et al., 2003, 2006; Dehaene and Changeux, 2005, 2011).

In what follows we specifically focus on large-scale brain simulation, discussing whether and how much it might improve our understanding of DOCs and their clinical management.

Different research projects have tried or are still trying to implement a large-scale brain simulation in recent years (de Garis et al., 2010; Markram, 2011; Serban, 2017). The specific goals and methodologies of these projects significantly vary (e.g., generating new data for better understanding specific phenomena or contributing to develop new theoretical models for improving neuroscientific knowledge; bottom-up integration of data vs. top-down approaches, etc.), while they share the attempt to simulate the brain on a large-scale, usually at specific levels and possibly including additional levels of biological descriptions in the model (Markram, 2011). Several conceptual and methodological challenges have been discussed concerning these attempts (Eliasmith and Trujillo, 2014; Milkowski, 2016; Colombo, 2017; Serban, 2017). An analytical assessment of these criticisms is beyond the scope of this paper, which instead refers to large-scale brain simulations trying to check whether they are relevant and useful for a better understanding and assessment of DOCs.

Very briefly, large-scale brain simulations can be described as a subclass of computer simulation.

Narrowly, computer simulation can be described as the use of a computer to solve an analytically unsolvable equation (Humphreys, 2009; Frigg and Reiss, 2009; Winsberg, 2009). Broadly, computer simulation can be equated to the entire process of developing, using and justifying a model that involves mathematics that is not analytically tractable (Frigg and Reiss, 2009). Three main features of computer simulations so defined emerge (Winsberg, 2009; Serban, 2017): they depend on particular implementation constrains; they rely on particular theoretical models; they play a justificatory role in drawing inferences about the target-object. Critical for the last characteristic are evaluation criteria of verification and/or validation.

The concept of large-scale brain simulation varies according to how “large-scale” is defined (de Garis et al., 2010). What mainly makes the difference is the amount of biological fidelity the modeler tries to include in the simulation. Accordingly, de Garis et al. (2010, p. 4) distinguish five different types of simulation and related goals, going from the highest to the lowest level of biological fidelity:

(1). Creating models that can actually be connected to parts of the human brain or body, possibly serving the same role as the simulated brain system;
(2). Creating a detailed functional simulation of a brain subsystem, i.e., a simulation of dynamics and inputs/outputs chains;
(3). Creating models that quantitatively simulate the dynamics of a brain subsystem without a precise functional simulation;
(4). Creating models that simulate brain subsystems or whole brain at high level, skipping particular details and focusing on some overall properties;
(5). Creating models demonstrating the capacity of hardware to simulate large neural models without any claims about the match of the models in question to empirical neuroscience data.

Thus large-scale brain simulations can have different levels of biological fidelity mainly depending on the goal of the simulation itself. Furthermore, the amount of biological fidelity is directly linked to the choice of the specific level and scale where to simulate the brain.

Technically speaking, strategies for large-scale models and simulations of the brain at a certain level of analysis are feasible and have already been developed (de Garis et al., 2010; Serban, 2017). For instance, the European Human Brain Project (HBP) is developing workflows and modeling strategies for modeling the brain at different scales, to then put together or bridge the results obtained from the different levels of organization (Amunts et al., 2016). The HBP employs a data-driven strategy of “components models”: the research is intended to model a phenomenon at a certain scale, modeling all its different components and then aggregating them to determine what happens at the higher level. However, there are of course a number of challenges. One is presented by modeling the links between the different levels, i.e., a strategy for modeling and then simulating all the levels of a target object (e.g., the brain) together in runtime. The challenge is both scientific (we do not know exactly all the different levels involved and the connection between them) and technical (present technology is limited and unable to provide a sufficiently detailed inter- and multi-level simulation).

Besides the challenge arising from our limited understanding of exactly how the brain is organized which limits our ability to simulate its organization, there is the challenge to model what the brain does, particularly its ability to represent the world (e.g., through cognitive or emotional experiences). Many present models lack representational capacities, i.e., they do not give us cues on the representational power of our brain (Pennartz, 2015). This might simply be a question of computational limited resources and related constraints (Eliasmith and Trujillo, 2014), or it might be related to the lack of mathematics needed for model the brain’s representational ability, so that it would be necessary to develop a different paradigm to bridge the gap between neuronal and representational.

Furthermore, our actual knowledge of the brain is still limited, certainly not sufficient for implementing a large-scale simulation encompassing all the brain levels. The collection of data needed for a realistic large-scale brain simulation is particularly challenging for technical and procedural reasons (e.g., heterogeneity of data format). Moreover, the data sampling implied in actual simulation modeling approaches could miss relevant data (Dudai and Evers, 2014).

It is, however, likely that we will soon be able to simulate the whole human brain on a specific level, e.g., on a cellular level, in a meaningful and relevant way if data for model reconstruction are produced (Markram, 2011). Yet, the challenge is that to be more useful, a whole brain model needs to have some minimal representation of experimental data over multiple biological scales. Although not impossible in principle, to date this hasn’t been technically feasible.

Does the above imply that the attempt to achieve large-scale brain simulation is useless for simulating consciousness? We suggest not. In fact, within a top-down approach (i.e., focusing on global, emerging dynamics of the brain, and starting from target behaviors or properties implementing elements and interactions that enable them), the simulation would be able to simulate the macro-level organization and behavior of the whole brain (as long as they are adequately simplified). This kind of large-scale brain simulation might be able to approximately simulate the brain dynamics during conscious tasks and then generate new hypotheses to be investigated further, even if the key question remains, namely: how to capture phenomenology, i.e., the qualitative dimensions of conscious experience.

Yet another strategy for simulating the whole-brain is possible; namely, an agent-based simulation (i.e., a simulation that generates the brain’s dynamics by calculating the dynamics of the constituent parts and then aggregating them) implementing a bottom-up approach (i.e., there is no starting behavior or property to be modeled but the modeler focuses on what arises from the interaction among the model’s components). Even in this case the specific target of the simulation (discrete brain function/structure) is relatively idealized. Furthermore, the general target (the whole brain) is divided in structural and functional sub-components to be prospectively aggregated in order to simulate the whole brain. When referring to conscious processing, this strategy has the procedural advantage of dividing a complex problem into more manageable parts: the different steps implemented within a r-up approach can be very relevant in order to model and simulate specific aspects of consciousness, especially when it comes to integrate and interpret experimental data. Another possible strategy is to build ‘hybrid’ models where certain brain networks are simulated in much greater detail (e.g., on the detailed cellular level) and then embed these model modules with the other model parts that are much more phenomenological.

We suggest that a mix of the two strategies (i.e., top-down and bottom-up, focusing on global brain dynamics or focusing on discrete brain components and then aggregating them) can be useful, resulting in a top-down/bottom-up multilevel approach. Such a hybrid strategy would entail starting from a theoretical description of the behavior or property to model (e.g., a visual conscious process), proceeding to divide this behavior/property into sub-components, and then determining whether actual simulation approaches that focus on specific brain structure/function are relevant for simulating specific components of the conscious brain as well.

In fact, the already mentioned technical and theoretical limitations might suggest a discretional approach to conscious brain simulation, one that aims at identifying specific relevant cerebral functions and structures.

## The Challenge to Simulate Consciousness

Two elements are critical for assessing the adequate scale (i.e., level of detail) of a model: the target object and the available computational technology (Gates, 1992; Eliasmith and Trujillo, 2014). In the case of consciousness, a first main obstacle to the attempt of simulating it is the lack of a shared definition of the target object. Generally speaking, one can investigate consciousness through simulating NCCs unless some form of dualism is true. Specifically, if operationalised in terms of NCC, it is theoretically plausible to investigate consciousness through simulating NCC.

Furthermore, even if defined in terms of NCC, consciousness can be differently described. Specifically, the main distinction between access and phenomenal consciousness is relevant to our discourse. Whether access and phenomenal consciousness are really two different forms of consciousness or rather two different aspects of the same underlying conscious activity continues to be debated (Pennartz, 2015). Still, it is commonly agreed that while access consciousness refers to the interaction between different states, particularly the availability of one state’s content for use in reasoning and in rationally guiding speech and action, phenomenal consciousness is the subjective feeling of a particular experience, “what it is like to be” in a particular state (Block, 1995).

Also, the already mentioned clinical/operational distinction between two components of consciousness, i.e., level (wakefulness) and content (awareness) (Laureys, 2005), is relevant in order to assess the possibility of simulating consciousness.

Another possibility, which has been very useful in empirical research, is to describe consciousness in terms of conscious access of information (Dehaene and Changeux, 2011). The contribution of whole-brain models in understanding information processing both in the resting and in the active brain has recently been outlined (Deco et al., 2015).

A very attractive way of simulating consciousness is by computer simulation, i.e., a computer-based implementation of mathematical models (i.e., software) on appropriate hardware in order to have a dynamic reconstruction of the brain’s (conscious) activity. Both computer simulation and typical neuroscience experiments suffer from the possible lack of factors that might be relevant to the investigation’s goals, e.g., neuromodulation present in the real system, or they can have a slightly wrong temperature, pH and ionic composition. This can result in some dynamics (e.g., synaptic dynamics) different from the real brain system *in vivo*. Yet this epistemic imbalance, i.e., the potential discrepancy between our experimental models or computer simulation and the actual brain, is an unavoidable feature of our current neuroscientific knowledge, which is always technically and theoretically mediated. Our knowledge of the brain is always mediated by the technology available and is always indirect in the sense that it focuses on features and factors we think are relevant on the basis of specific theoretical models.

In the case of consciousness, thus far simulation is defined as an integrative approach to test how available data and knowledge may explain phenomena considered indicative of conscious activity (e.g., synchronization of the neuronal activity in specific cortical areas). This kind of simulation has been widely and successfully applied to specific neuronal components of conscious activities (Dehaene et al., 2003; Dehaene and Changeux, 2005), so that we can legitimately conclude that identified NCC (of both contents and states) can realistically be modeled though computer simulation.

The situation can be trickier if no specific NCC is identified, or if consciousness correlates with more than one specific brain subcomponent, or if consciousness is considered as emerging from the brain as a whole system.

For instance, the prediction of the properties of the brain as a whole on the basis of the properties of single components (e.g., electro-chemical properties) is not always possible, for at least three main reasons (Roth, 2013). First the brain is a highly complex organ; second, available mathematics is strongly limited and fundamentally unable to deal with qualitative properties; and finally most components inside the brain change their properties while interacting. Among other things, this means that:

(a) The brain is far more than an input-output machine. It can be described as a network with hidden internal layers, and its activity between the input and output layer [which seems critical for consciousness and peculiar to conscious systems as opposed to artificial ones (Tononi and Koch, 2015)], often cannot be precisely reconstructed mathematically. Notwithstanding the important achievements in the investigation of the resting state of the brain and of its intrinsic activity, much remains to be done for getting knowledge and generate data that could be sufficient for simulating it.
(b) At the local level, the properties of the brain components are relatively changeable depending on their reciprocal interaction. Modeling a single component is not sufficient to get a reliable prediction of its behavior: the reciprocal interaction of the different components and their new resulting properties should be modeled as well. Even if deterministic in their development, these factors (interactions and resulting properties) are highly stochastic, i.e., appear random. A simulation of stochastic systems and their internal or external interactions can only be formal in the sense that we can simulate a possible dynamics on the basis of the extracted regularities (i.e., general principles or fundamental rules of organization and development). This would result in a type simulation (i.e., construction of a typical, ideal object), not in a token simulation (i.e., simulation of a specific real object). However, type simulation would be an important achievement: an ideal, typical brain could be very useful to infer properties of individual real brains. Furthermore, searching for indirect knowledge (from the ideal to real brains) seems to be a necessary epistemological strategy, at least today. The hope is to discover the principles underlying the internal brain organization that emerges as a result of activity (both intrinsic, i.e., independent from external stimuli, and extrinsic, i.e., dependent on external stimuli) so that we can train an ‘ideal generic brain’ to become functional and thus develop into a ‘specific time-defined individual brain’.
(c) At the global level the brain exhibits properties and functions that supervene its different, particular components.

The abovementioned limitations might result in a critical impasse of any attempt to simulate consciousness, unless we identify specific NCC for it. However, even in this case, what seems plausible to model and eventually simulate is not consciousness as such, but only the cerebral configuration (i.e., ‘neural correlates’) likely related to consciousness. For instance, a main limitation of neuronal networks model is their inability to “modally” identify or recognize the inputs they receive: they are basically organized in functional terms, i.e., from an input to an output that may represent anything (Pennartz, 2015). According to Pennartz, this difficulty depends on the fact that the models are underconstrained or not detailed enough (Pennartz, 2015). Particularly, Pennartz outlines that if the aim is to model phenomenal conscious contents, we must keep in mind that phenomenal perception cannot be explained at the level of neurons or networks of neurons, but rather at the level of networks of neurons’ ensembles, i.e., of groups of neurons located in the same sub-region and operating in a similar way. The point is that the brain has a multilevel organization (i.e., molecular level, gene- or signaling networks, neurons, neurons’ ensembles, unimodal and multimodal networks of ensembles), and the relation between the different levels is not in terms of one-way causality, but in terms of reciprocal causality. Moreover, the different brain levels refer to the same phenomenon (e.g., conscious perception) from different perspectives: during a conscious perception something happens simultaneously at different levels. If the goal is to model phenomenal conscious perception the focus should be on the multimodal networks of ensembles. Still, the resulting simulation would be simulation of perception not as an experience, but as a cerebral dynamics. The conceptual gap between neuronal activity and perception seems hard to bridge.

(d) In its basic form, primary consciousness has been provocatively proposed to be a simulation-based interaction with the external environment (Merker, 2007; Barron and Klein, 2016): subjective experience is grounded on the midbrain, which produces an integrated simulation of the state of subject’s own mobile body within the environment. This simple form of consciousness is the basis for more complex human forms of consciousness, like self-reflexive consciousness, access consciousness, and higher-order awareness. Merker suggests that the midbrain plays an important role in producing this simulation: it combines interoceptive (stimuli arising from within the body) and exteroceptive (stimuli external to the body) sensory information. The simulation produced by the midbrain is constructed from appropriate integration of afferent, efferent, and homeostatic information (Roth, 2013). In other words, the basic form that allows any other form of consciousness is grounded on a neural modeling of the subject’s own body and the external space.

A simulation view of consciousness and self-consciousness has been elaborated also in philosophy. For instance, this view is central in some important contributions by Metzinger (2000c, 2003, 2009). The self-model theory of subjectivity suggested by Metzinger is grounded on what he calls the phenomenal self-model, the conscious model of the organism as a whole activated by the brain (Metzinger, 2009). The core idea is that consciousness and self-consciousness are simulation processes, which are transparent, i.e., we are not aware that our consciousness of the world and of ourselves is a model that our brain builds. More specifically, “First, our brains generate a world-simulation, so perfect that we do not recognize it as an image in our minds. Then, they generate an inner image of ourselves as a whole” (Metzinger, 2009) (p. 6). Being a simulation, consciousness is a highly selective representation, so that Metzinger qualifies it as a tunnel, which results from the information flow in the global NCC.

Similar views are expressed by Antti Revonsuo, who uses “world simulation metaphor” to outline that we are not really in contact with the external world, but only with an internal phenomenal world, i.e., with a phenomenal model of the external world (Revonsuo, 2006). For this reason Revonsuo says that there is a virtual reality inside the brain (Revonsuo, 2010).

Given the above summarized description of consciousness as a simulation suggested by both neuroscience and philosophy, to simulate the conscious brain means to simulate a simulating system, resulting in a kind of second order simulation (or metasimulation).

## Potential Applications to Disorders of Consciousness (DOCs)

Recent studies show residual metabolic and electrophysiological activation in some cortical areas in patients with DOCs (Schiff et al., 2002). Notably one patient behaviourally diagnosed with unresponsive wakefulness syndrome showed a cortical activity in specific areas similar to healthy controls (Owen and Coleman, 2008). Significantly many behaviourally conscious patients failed the same test (Monti et al., 2010). From this we can legitimately infer that, as said before, NCCs are not sufficient for explaining consciousness: it is too complex to be reduced to NCCs. Consequently, simulating NCCs is not the same as simulating consciousness. For doing so we need further advancement in the development of an appropriate theory of consciousness.

Notwithstanding this and the other above-mentioned conceptual and technical difficulties, it is possible to simulate specific components of consciousness both within a large-scale approach calibrated on a specific level/scale or within a discrete approach. Moreover, such simulation is potentially useful in clinical contexts, specifically in the assessment and care of patients with DOCs.

There are important potential advantages that seem to be relevant for the particular simulation of consciousness and its disorders (Markram, 2013):

– no limit on what we can record, i.e., we can obtain a potentially unlimited amount of data from a simulation (as everything in the model is measurable).
– no limit on the number of manipulations we can perform (i.e., all model parameters can be manipulated).
– enhanced replicability and interpretation of experiments.
– the possibility of building bridges between different levels of brain organization (i.e., the possibility of understanding the relative correlation between different space and time scales within the brain).
– the possibility to simulate brain diseases with major clinical diagnostic, prognostic, and possibly therapeutic implications.

All the above-mentioned points are particularly relevant for a better diagnosis and prognosis of DOCs. When people are unable to explicitly express their conscious state, directly exploring the condition of the detected brain signatures or correlates of consciousness is critical. This assessment is presently performed through brain measurements and neuroimaging, e.g., Electroencephalogram (EEG), functional magnetic resonance imaging (fMRI), positron emission tomography (PET), which although highly informative can also eventually be misleading and affected by intrinsic constraints (Eklund et al., 2016). We suggest that computer simulation, ultimately combined with classical brain measurements and neuroimaging, particularly for verification and/or validation, might help overcome this limitation. Specifically, in the case of patients with DOCs whose involvement in neuroimaging measurements may be both technically and ethically challenging, a computer simulation could fill the gap of missing data, or give clinicians the tool for predicting the future development of a disorder or the outcome of a particular treatment. Furthermore, it is theoretically possible to simulate different scenarios (e.g., different medications) through a computer simulation, particularly the effects of treatment at different brain levels (e.g., molecular, neuronal, and synaptic) and at the intersection of different levels. It is also possible to manipulate and to replicate experiments in order to get the most informative data on the patients’ present and future conditions. In this way, simulation may allow more informed decisions about the patients’ treatment.

In short, through brain simulation we may prospectively overcome the present fragmentation of our knowledge of the brain, speeding up progress in our knowledge development, with important consequences for our understanding of the conscious brain in general and of DOCs in particular, including the possible development of new, more effective diagnostic, prognostic and therapeutic protocols. First, it is likely that computer simulation will allow us to simulate specific diseases of the brain at different scales of complexity, which could be extremely useful for pre-clinical studies. Second, simulation can give us data complementary to empirical and behavioral observation, and thus allow us to make tests and predictions that are not possible either *in vivo* or *in vitro*. Third, simulation can allow us to predict and infer general rules describing the organization of particular brain levels (e.g., neocortex at the cellular level) (Markram et al., 2015).

It might be that the scale of identified consciousness’ subcomponents (e.g., of wakefulness and awareness) is still too large for the available simulation technology, but they can in principle be simulated through a bottom-up approach in order to predict their pathological development or to test possible therapeutic strategies. For instance, we could develop a mechanistic model of the whole brain at a certain level (e.g., cellular level), and then manipulate the model to simulate effects like anesthesia or other medications.

Thus, in spite of the present conceptual and technical challenges that we have here described, computational models and simulations seem already in a position to offer new tools to implement ordinary or experimental treatments with DOC patients. This may have important clinical implications, both in terms of better knowledge and in terms of reducing the risks of these patients’ direct enrolment in research.

## Conclusion

Simulation of consciousness through large-scale brain models seems to be possible in principle, even if present tools for modeling are limited (e.g., mathematics) and our current insufficient understanding of the brain’s structure and the functional neural code of consciousness makes such simulation technically and conceptually limited. A mix between a top-down and a bottom-up approach might be a reasonable strategy to implement: starting from a target phenomenon to be modeled (e.g., a particular conscious perception) and then discretizing the whole brain into functional and structural subcomponents(top-down); and developing a discrete description of some of these subcomponents to explain their function and predict their development (bottom-up).

While simulating all the levels of the conscious brain simultaneously appears presently far-fetched, the simulation of specific NCCs both within large-scale and discrete approaches seems a more feasible goal, with potential scientific as well as clinical implications, as illustrated by the case of DOCs. There is a scientific as well as a conceptual need to continue developing instruments (tools and software) that may better integrate the presently fragmented knowledge of the different levels of organization of the brain, i.e., the microscopic and the macroscopic levels, allowing us to bridge basic biological with higher cognitive functions, and maybe, with time, bridge the apparent neuronal-phenomenological gap.

## Author Contributions

MF wrote the manuscript and was responsible for general ideas. KE contributed to revising and developing ideas. KE and JK commented on previous versions of the manuscript and helped in developing lines of argument.

## Conflict of Interest Statement

The authors declare that the research was conducted in the absence of any commercial or financial relationships that could be construed as a potential conflict of interest.
